# Role of Actin Dependent Nuclear Deformation in Regulating Early Gene Expression

**DOI:** 10.1371/journal.pone.0053031

**Published:** 2012-12-28

**Authors:** Soumya Gupta, Nimi Marcel, Apurva Sarin, G. V. Shivashankar

**Affiliations:** 1 National Centre for Biological Sciences, Tata Institute of Fundamental Research, Bangalore, India; 2 Department of Health Sciences, Manipal University, Manipal, Karnataka, India; 3 MechanoBiology Institute & Department of Biological Sciences, National University of Singapore, Singapore, Singapore; University of Iowa, United States of America

## Abstract

The nucleus of a living cell is constantly undergoing changes in shape and size as a result of various mechanical forces in physiology. These changes correlate with alterations in gene expression, however it is unclear whether nuclear deformation alone is sufficient to elicit these alterations. We used T-cell activation as a model system to test the coupling between nuclear deformation (elongation) and gene expression. Naïve T-cell activation with surrogate antigens resulted in actin dependent nuclear elongation. This was accompanied with Erk and NF-κB signaling to the nucleus to induce CD69 expression. Importantly, inhibiting actin polymerization abolished both nuclear elongation and CD69 expression, while inhibiting Erk, NF-κB or microtubule depolymerization only abolished expression but not elongation. Immobilization of antigen-coated beads, under conditions where actin polymerization was inhibited, rescued both nuclear elongation and CD69 expression. In addition, fibroblast cells plated on fibronectin micropatterns of different sizes showed correlation between nuclear shape index and tenascin C expression. Upon inhibiting the signaling intermediate Erk, tenascin C expression was down regulated although the nuclear shape index remained unaltered. Our results highlight the importance of specific signaling intermediates accompanied with nuclear deformation in the modulation of cellular genomic programs.

## Introduction

The cell nucleus is subjected to changes in shape and size during several physiological processes, such as differentiation, ageing and disease [1]. Embryonic stem cells have a spherical nucleus, which generally becomes ellipsoid as they differentiate [2]. Drosophila embryos also have round nuclei during initial stages of development, but during germ band extension and with the onset of cellularization, the nuclei become elongated. The 2.5-fold increase in length of these nuclei coincides with chromocentre formation and expression of developmental genes [2]. Bone marrow progenitors differentiate into neutrophils that acquire multi-lobed nuclei, thus allowing easy inter-cellular transmigration [3]. Furthermore, several studies have demonstrated that cell geometry can impinge on nuclear shapes and sizes and chromatin remodeling [4]–[6]. Altering nuclear shape by plating cells on micro-fabricated substrates correlated with changes in cytoskeletal reorganization and gene expression [7], [8].

Nuclear lamins that link the nuclear membrane with chromatin, and cytoskeletal proteins that link the plasma membrane with the nuclear membrane control nuclear shape and size [5], [9]. Depolymerization of actin filaments decreases nuclear size, whereas depolymerization of the microtubule network increases nuclear size [10]. Lamin A deficient cells had increased nuclear deformation and poor viability in response to mechanical strain [11]. NF-κB and cytokine mediated gene expression was also less in these cells compared to wild type fibroblasts suggesting altered transcriptional programs. On the other hand, emerin mutant fibroblasts had an altered nuclear shape but normal nuclear mechanics [12]. These mutant cells also displayed impaired expression of mechanosensitive genes. Nuclear shape changes may alter transcription factor affinity for DNA, transport to the nucleus, nuclear matrix organization and the microenvironment of genes by bringing them closer to or further away from transcription factories and other active/repressive genes [8]. However, it is still not clear whether changes in nuclear morphology alone are sufficient to induce changes in gene expression.

T-cell activation is a good model system to study the effects of nuclear shape on gene expression as their activation is followed by actin-mediated cell polarization resulting in nuclear elongation, as well as the induction of early activation genes (EAGs). Perturbation of actin polymerization inhibited nuclear elongation and EAG expression. On the other hand, perturbation of signaling intermediates (Erk, NF-κB) only perturbed gene expression but not nuclear elongation. Additional experiments are presented using NIH3T3 fibroblast cells cultured on fibronectin micropatterns of three different contact areas. The correlation between nuclear shape index (NSI) (nuclear area/height) and Tenascin C expression is also presented. These results suggest that nuclear deformation alone is not sufficient for gene expression, but acts in concert with signaling intermediates in various physiological contexts to induce the expression of appropriate genes.

## Materials and Methods

### Ethics Statement

All experiments involving animals were performed with the approval of the Institutional Animal Ethics Committee at NCBS, Bangalore headed by Prof. Mathew with the help of Professors Upinder Bhalla, Sumantra Chatterjee, MM Panicker and R. Sowdhamini. Approval ID for the project is AS-5/1/2008.

### Mice, Cells and Activation

All experiments used C57/Bl6 mice or transgenic B6.Cg-Tg (Hist1H2BB/EGFP) mice. The H2B-EGFP transgenic mouse was purchased from The Jackson laboratory (http://jaxmice.jax.org). These mice express the H2B protein fused to EGFP under the chicken beta-actin promoter coupled to CMV immediate early promoter/enhancer. As a result all nucleated cells in the mouse have fluorescence. CD4^+^ naïve T-cells were isolated from spleen of 8–10 week old mice using MagCellect isolation kit (R&D Systems, MN). 1×10^5^ cells were stimulated with antigen coated beads (Invitrogen, CA) in 24-multiwell plates at a stoichiometry of 1∶2 (cell:bead) before staining for CD69 or CD25. For bead immobilization, plates were placed on a permanent magnet. Distance between plate and magnet was varied to modulate the force on bead. To vary the time of exposure to force, plates were removed from the magnet at the indicated times and maintained in culture for 3 hours, followed by expression analysis.

### Drug Treatments

For all drugs, cells were pre-treated for 30 minutes prior to plating along with the antigen-coated beads and stained for CD69 after 3 hours. For calculation of nuclear shape, cells were imaged 30 minutes after plating. Drug concentrations used were as follows: IKK2 inhibitor 5 µM, Cytochalasin D 2 µM, Nocodazole 1 µg/ml, Latrunculin A 1 µM, Blebbistatin 15 µM, U0126 10 µM. Inhibitors were purchased from Calbiochem (Germany) and Sigma Aldrich (MO). For drug treatment in NIH3T3 fibroblasts, cells were treated with 25 µM U0126 for 15 minutes, plated on micropatterns in presence of the drug, and stained after 4 hours of incubation.

### Immunostaining

For staining, cells were activated on poly-d-lysine coated glass bottom dishes, fixed with 1% PFA for 20 minutes, permeabilized with 0.2% NP-40 for 5 minutes and blocked with 10 mg/ml BSA for 1 hour at room temperature. Cells were sequentially incubated with primary and secondary antibodies. Antibody for phosphorylated Src Tyr-416 was from Millipore (Germany), Tenascin C Ab from Abcam and secondary antibodies from Invitrogen (CA). Actin was stained using rhodamine phalloidin and DNA with Hoechst 33342 (Sigma).

### Flow Cytometry

Post-stimulation cells were harvested, beads removed and washed with FACS buffer (PBS with 1% FBS and 0.1% NaN_3_) followed by incubation with αCD69-PE, αCD25-PECy5 or αThy1.2-FITC (eBioscience, CA, 1∶100 dilution) on ice for 30 minutes. Cells were washed again, resuspended in FACS buffer and analyzed by flow cytometry. All flow data was acquired on Cyan (Dako) flow cytometer.

### Confocal Imaging

Zeiss (LSM510-Meta) or Olympus FV1000 confocal fluorescence microscopes were used for imaging experiments using 63×/1.4 N.A oil immersion objective. 8-bit images of 512-by-512 pixels were acquired with 1 airy-unit pinhole aperture size and a z-step size of 500 nm. Time-lapse live cell images were recorded at 5 seconds/frame, with stage temperature maintained at 37°C and CO_2_ concentration maintained at 5%. 488 nm laser line was used to excite EGFP or Alexa 488 tagged proteins, 543 nm for rhodamine phalloidin and 405 nm for Hoechst 33342.

### Microcontact Printing

Silicon wafers with the desired designs were coated with silane (Sigma Aldrich, MO) after which PDMS elastomer (Sylgard 184, Dow Corning) at ratio of 1∶10 of curative to precursor was poured onto the wafer and cured at 80°C for 2 hours. After the PDMS was set, stamps were cut, plasma treated, and coated with 100 µg/ml fibronectin solution. After 5 minutes, excess solution was drained with tissue paper, and stamp was allowed to air dry for 5 minutes. The stamp was then gently inverted onto a 35 mm plastic bottom dish (Ibidi, Germany), allowed to sit for 2 minutes and then removed. The dishes were coated with 0.2% pluronic for 15 minutes, washed with PBS and seeded with 30,000 NIH3T3 cells in 2 ml medium. Extra cells were removed by washing with medium after 45 minutes of plating. At the end of four hours, cells were stained with α-Tenascin C antibody (1∶350 dilution) and imaged.

### Western Blotting

Whole cell lysates for 1×10^6^ cells were resolved by SDS page and analyzed with standard immunoblotting protocol. Phosphorylated Erk antibody (Thr202/Tyr204), phosphorylated p130Cas (Tyr165) (used at 1∶500 dilution) and secondary antibodies were from CST (MA). Blots were probed with α-tubulin (Neomarkers, CA) for normalization.

### Real-time PCR

RNA was isolated from CD4+ naïve, M− and M+ activated T-cells. Equal amounts of RNA were used for cDNA synthesis using M−MuLV Reverse Transcriptase (Invitrogen, CA). Real time PCR was carried out with SYBR Green qPCR Mastermix (SABiosciences) using ROTOR-GENE RG 3000 (Corbett Research). PCR was carried out in duplicates and average Ct value was calculated for CD69. 18S rRNA was used as loading control.

### Data Analysis

Image J was used for all image analysis. Nuclear aspect ratio was calculated as a ratio of major axis to minor axis of the nucleus and data was binned into histograms. Only cells that made contact with beads were considered for this analysis. Centroid tracking of beads and cells was done using MTrackJ plugin. Statistical analysis for all data in bar graphs is presented as mean±SD or mean±SE derived from a minimum of three independent experiments. Statistical significance was calculated using a two-population student t-test.

## Results and Discussion

### Nuclear and Cellular Remodeling Accompany Expression of Early Activation Genes

**Figure 1:**
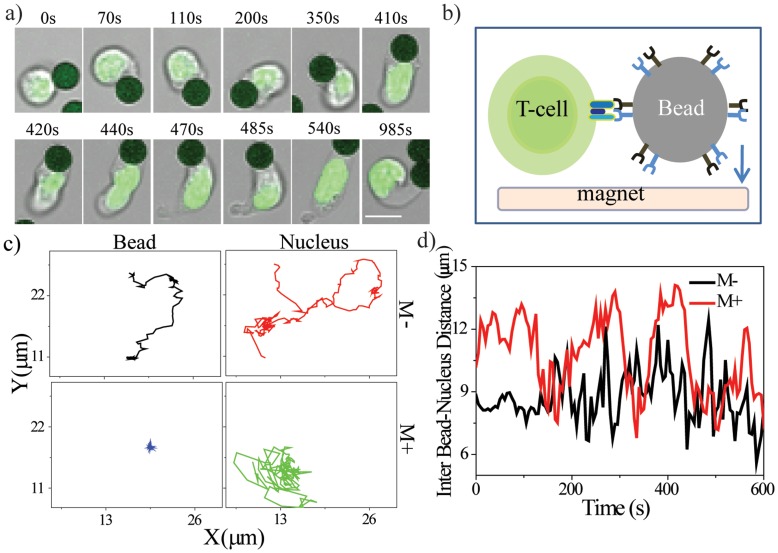
Nuclear and cellular remodeling accompany T-cell activation. a) Representative time-lapse images of T-cells during activation. Green- H2B EGFP nucleus; black- antigen-coated beads. Scale bar 5 µm. b) Schematic representation of experimental design. c) XY trajectory of the centroid of a bead and cell nucleus in M− and M+ conditions. d) Distance between the centroids of a bead and a cell nucleus in M− and M+ conditions.

*In vitro* activation of T-cells with surrogate antigens, αCD3-αCD28, has been shown to induce localization of membrane proteins and cytoskeleton at the T-cell receptor (TCR) complex, resulting in cell polarization [13]. Naïve T-cells derived from H2B-EGFP transgenic mice were activated with 4.5 µm sized paramagnetic beads coated with surrogate antigens and time-lapse images were recorded. Figure 1a shows time-lapse images of bead-T-cell contact resulting in rapid changes (within a few hundreds of seconds) in cellular and nuclear morphology, a process central to T-cell activation. Immuno-staining for phosphorylated Src (pSrc) protein showed enhanced intensity at the cell-bead junction (figure S1), consistent with previous literature [14]. To mimic the *in vivo* process of activation, where antigen presenting cells (APCs) are immobile and present in dense tissue spaces [15], [16], αCD3-αCD28 coated beads were immobilized using a permanent magnet (figure 1b) with ∼324pN force (figure S1). Time-lapse movies for both conditions were recorded: in the absence (M−) and presence of a magnet (M+) (Movies S1 and S2). Particle tracking analysis of centroid positions of the cell nucleus and bead in either condition is shown in figure 1c. As seen in the XY trajectories, beads that contacted cells were displaced from their original positions in the M− condition but not in the M+ condition, while trajectories for nuclear centroid positions in both conditions remain unchanged. Interestingly, the centre-centre distance between nucleus and bead (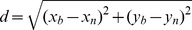 ) shows a cyclic displacement pattern (figure 1d). The peak of displacement appeared to be higher in the M+ condition than in the M− condition, suggesting generation of greater force. These experiments recapitulated the characteristic nuclear elongation (deformation) associated with T-cell activation.

**Figure 2:**
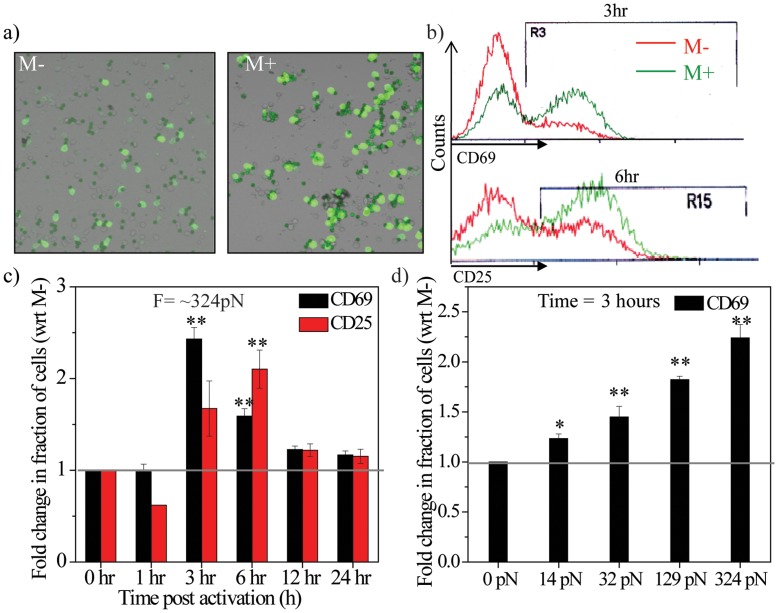
Expression of early activation genes is modulated by signal presentation. a) Images of CD69 stained T-cells in M− and M+ conditions. Green- CD69, Greenish black- antigen-coated beads. b) Flow cytometry analysis of CD69 and CD25 in the M− and M+ conditions at 3 and 6 hr, respectively. c) Mean fold change in CD69 and CD25 expressing cells in the M+ condition with respect to the M− condition at different time points (N = 3, mean±s.e.m.). **p<0.005. Statistical significance is calculated with respect to the M− sample at each time point. d) Mean fold change in CD69 expressing cells at 3 hr in the M+ condition (with different bead immobilizing forces) with respect to the M− condition (0 pN) (N = 3, mean±s.d.). *p<0.05, **p<0.005. Statistical significance is calculated with respect to 0pN force.

In addition to nuclear elongation, T-cell activation results in the induction of EAGs [17]–[19]. Figure 2a shows immunofluorescence images of CD69 expression in M− and M+ conditions. Cells were activated using antigen-coated beads and flow cytometry analysis carried out to assess the expression of EAGs in both conditions (figure 2b). Interestingly, the fraction of CD69 positive cells at 3 hours was higher in the M+ condition compared to the M− condition (50% and 17%, respectively). Similarly, CD25 positive cells at 6 hours were higher in the M+ condition compared to the M− condition (43% and 21%, respectively). Figure S1 shows the fraction of cells positive for CD69 or CD25 expression in both conditions at various time intervals post-activation. Notably Mean Fluorescence Intensity (MFI) of protein expression per cell was similar (figure S2). Incubation of T-cells with similar sized but non-specific magnetic beads resulted in negligible induction in both conditions, suggesting that TCR stimulation was necessary for the up-regulation of EAGs (figure S2). In addition, expression of an unrelated gene Thy1.2 was unchanged in both conditions, showing that the induction process was specific to EAGs (figure S2). We ensured that differential changes in gene expression were not a consequence of the density of cell-bead contacts, as in both conditions the fraction of bead-contacted cells were similar (figure S2).

Figure 2c shows the time-course kinetics of CD25 and CD69 positive cells. The fold change (M+/M−) in the fraction of cells expressing CD69 showed a maximal change at 3 hours, while for CD25 it was at 6 hours. At later time points the change was lower, suggesting modulation of gene expression at early time points (figure 2c, S1). Experiments were carried out at varying strengths of bead immobilization to test if cell-bead junction stability had a role in CD69 expression. In order to achieve this, distance between the culture dish and magnet was varied (figure S1b). The fraction of cells that stained positively for CD69 was shown to increase when the force (0–324 pN) applied on the beads was increased (figure 2d). Figure S2 shows the role of exposure time on CD69 expression. Interestingly, cells exposed for a longer time exhibit a higher fraction of CD69 positive cells. These results suggest that bead immobilization enhances the expression of EAGs in a manner that is dependent on both the magnitude and duration of bead immobilization. Figure 1 and 2 therefore establish that elongation of the nucleus is accompanied with changes in gene expression. We next tested the role of cytoskeletal remodeling in nuclear elongation and its coupling to early gene expression.

### Nuclear Elongation is Dependent on Actin Remodeling

**Figure 3:**
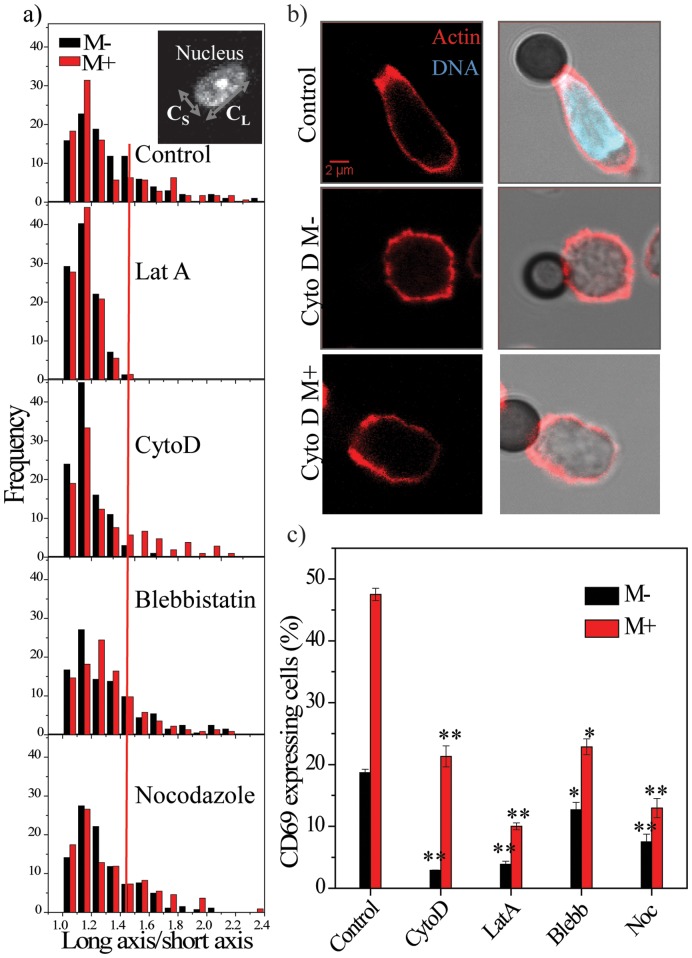
Nuclear elongation is dependent on actin remodeling. a) Normalized histogram showing C_L_/C_S_ values in activated T-cells in M− and M+ conditions in control or after treatment with various inhibitors (n = 200). The red line is used as a cut-off that marks round cells. Inset shows image of a nucleus depicting C_L_/C_S_. b) Actin, DNA and bright-field images for control and cytochalasin D (cytoD) treated M− and M+ cells. In the bright-field image, black represents the antibody-coated beads. Scale bar 2 µm. c) Fraction of cells positive for CD69 in control or cells treated with cytoskeletal inhibitors. *p<0.05, **p<0.005. Statistical significance is calculated with respect to control samples (M− or M+).

Cytoskeletal proteins are directly or indirectly linked to the nuclear membrane and influence nuclear organization and gene expression [9], [20]. The T-cell nucleus is elongated during activation as shown in figure 1. Inhibitors of actin and microtubule polymerization and actomyosin contractility were used to test role of cytoskeletal proteins in nuclear elongation and gene expression. Nuclear elongation was compared in control and inhibitor treated cells in M− and M+ conditions after 30 minutes of activation. Ratio of the long axis to short axis (C_L_/C_S_) was calculated for each nucleus (only in bead contacted cells) and representative histograms are shown (figure 3a); inset shows an image of a nucleus with arrows representing C_L_ and C_S_. Control cells showed elongated nuclei in both conditions and depolymerization of actin with latrunculin A (Lat A) resulted in round nuclei in both conditions (Figure 3a, S3). On the contrary cells treated with nocodazole, which depolymerizes microtubules, or blebbistatin, which inhibits actomyosin contractility, exhibited elongated nuclei (figure 3a, S3). This suggests that actin polymerization was a major determinant of nuclear elongation. Reinforcing this, treatment with cytochalasin D (cytoD) showed elongated nuclei in the M+ condition but not in the M− condition (figure 3a, S3), suggesting that reduced actin dynamics were enough to induce elongation of the nucleus if the beads were immobilized. Supporting this, phalloidin stains revealed enrichment of actin at cell-bead junction in control untreated cells (figure 3b). This enrichment was absent in cytoD treated cells in the M− condition, but the cytoD-M+ condition partially rescued actin enrichment at cell-bead junctions (figure 3b).

We next tested the correlation between nuclear elongation and CD69 expression. Treating cells with nocodazole (Noc) or latrunculin A (LatA) almost completely inhibited expression (<10% positive cells) (figure 3c). However, treatment with blebbistatin (Blebb) resulted in only a partial decrease in expression in both conditions (12% and 20% positive cells for M− and M+ respectively). Interestingly, inhibition of actin polymerization using CytoD inhibited expression in the M− condition, but resulted in a partial rescue of expression in the M+ condition with ∼20% positive cells (figure 3c). These experiments suggest that while nuclear deformation is correlated with gene expression (rescue in cytoD-M+ condition), it alone is insufficient to drive expression of CD69 (as in the case of nocodazole treatment). Further experiments were aimed at testing the role of well-characterized signaling intermediates in nuclear elongation and CD69 gene expression.

### Role of Erk and NF-κB in Nuclear Elongation and CD69 Expression

Signaling molecules that are known to influence CD69 expression [21] were inhibited to evaluate their role in nuclear elongation and CD69 expression. Firstly, we established that the expression of CD69 gene was transcriptionally regulated. When cells were activated in presence of 5,6-dichlorobenzimidazole riboside (DRB), a transcription inhibitor, CD69 expression was down-regulated in both M− and M+ conditions to just 10% and 25% of cells, respectively and these cells displayed very low MFIs (figure S4). Transcriptional regulation of CD69 was also confirmed by real-time PCR. Naïve T-cells had low levels of CD69 mRNA, which increased ∼2-fold and ∼10-fold in M− and M+ samples 30 minutes post-activation, respectively (figure S4). Inhibition of protein synthesis by cycloheximide predictably resulted in complete inhibition of expression (figure S4).

**Figure 4:**
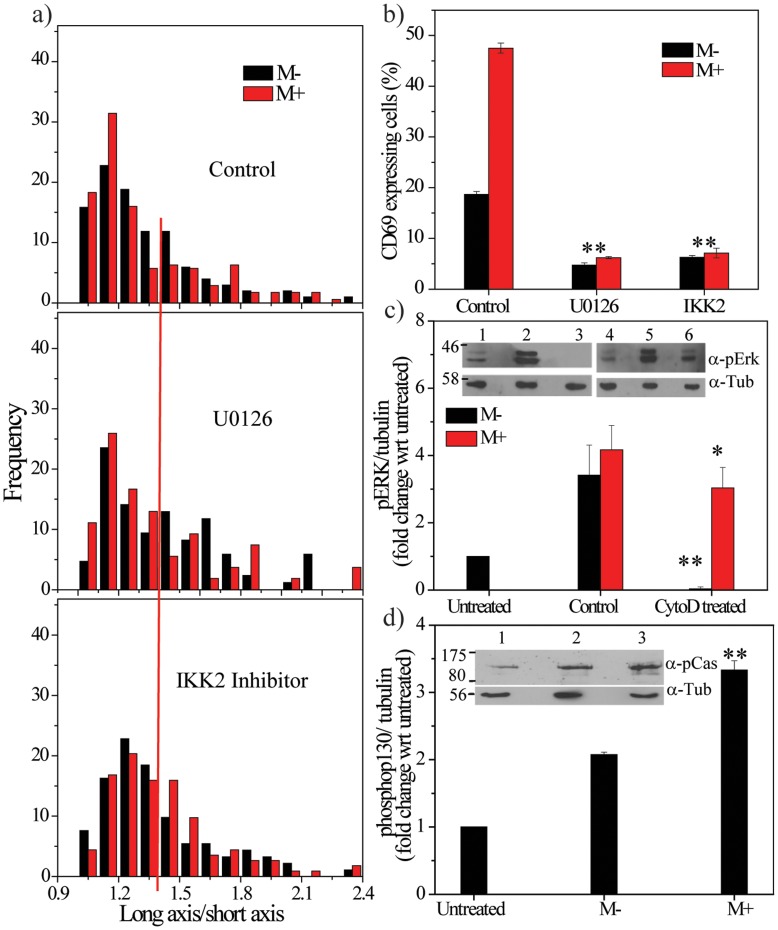
Role for Erk and NF-κB in nuclear elongation and CD69 expression. a) Normalized histogram showing C_L_/C_S_ values in activated T-cells in M− and M+ conditions in control and cells treated with U0126 or IKK2 inhibitors (n = 200). The red line is used as a cut-off that marks round cells. b) Fraction of cells positive for CD69 in control or in presence of U0126 or IKK2 inhibitors. **p<0.005. Statistical significance is calculated with respect to control samples (M− or M+). c) Fold-change in levels of pERK in T-cells in control (M− and M+) and cytoD treated (M− and M+) conditions (N = 3, mean±s.d.). Inset- representative Western blot for pErk (top) and tubulin (bottom). Lane 1: untreated; 2: M−; 3: M-cytoD+; 4: untreated; 5: M+; 6: M+cytoD+. Statistical significance is calculated for cytoD treated samples with respect to their corresponding control. *p<0.05; **p<0.005. c) Fold change in levels of phosphorylated p130Cas in M− and M+ conditions (N = 3, mean ±s.d.). Inset- representative Western blot for phosphorylated p130Cas (top) and tubulin (bottom). lane 1: untreated; 2: M−; 3: M+. Statistical significance is calculated with respect to M−. **p<0.005.

To test the coupling between Erk activation and nuclear elongation, cells were treated with the Erk inhibitor, U0126, and imaged after incubation with surrogate antigen-coated beads. Nuclear elongation remained unaffected in both M− and M+ conditions after blocking Erk activation (figure 4a, S3). Likewise, inhibition of the transcription factor NF-κB, speculated to regulate CD69 expression [21], with the IKK2 inhibitor, also had no effect on nuclear elongation (figure 4a, S3). Next, we tested the effect of these drugs on CD69 expression. Treatment of cells with either of these inhibitors resulted in complete shutdown of expression in both conditions, with expression only seen in ∼5% of the cells (figure 4b). These results show that signaling intermediates only affect gene expression but not nuclear elongation, further supporting the hypothesis that nuclear elongation alone is insufficient to induce gene expression. Since CD69 expression was rescued in the cytoD treated M+ condition, we tested if Erk activity (pErk) was also restored under these conditions. Western blotting analysis revealed ∼4-fold higher pErk in cells stimulated with TCR antibodies for 30 minutes (M− and M+ conditions) when compared to unstimulated cells. Interestingly, the cytoD M+ condition showed ∼3-fold higher pErk when compared to unstimulated cells, but the cytoD M− sample did not (figure 4c). These results show the rescue of nuclear elongation and signaling intermediates in cytoD treated cells when the beads were immobilized and suggests a role for both components in CD69 expression. Furthermore, we tested whether the strengthening of bead-cell junction could activate p130Cas, a protein sensitive to cell stretching [22]. In our experiments, we observed cyclic displacement between cell and bead (figure 1d), which may suggest that proteins can also be stretched in the cell in a cyclic manner, exposing them to phosphorylation events. Western blotting for phosphorylated p130Cas showed that the M− condition had ∼2-fold higher levels and M+ cells had ∼3.5-fold higher levels compared to unstimulated cells, within 15 minutes of TCR activation (figure 4d). Since TCR activation resulted in cell and nuclear deformation in T-cells, we next tested if nuclear deformation was sufficient to induce gene expression in other cell types. Towards this, experiments were conducted on fibroblast cells plated on micro-patterned substrates to modulate both cellular and nuclear deformation.

### Nuclear Deformation and Gene Expression by Modulating Cell Geometry

**Figure 5:**
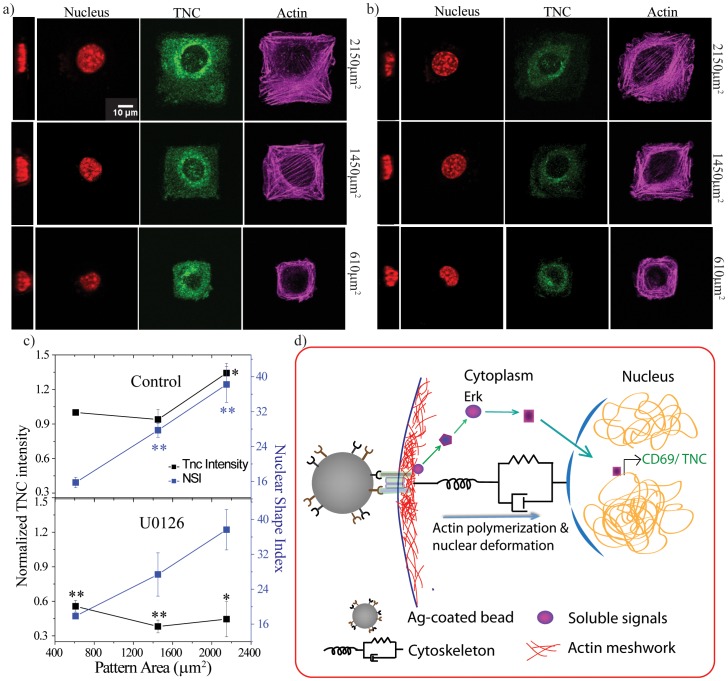
Nuclear deformation and gene expression by modulating cell geometry. Staining for nucleus, TNC and actin in control (a) and Erk inhibitor (b) treated cells on squares of different sizes. On extreme left of each panel x-z projection image of nucleus is shown. Scale bar 10 µm. c) Plot of integrated TNC intensity (normalized to smallest square of control for all samples) and NSI values for control (upper panel) and Erk inhibitor (lower panel). N = 3. Statistical significance calculated with respect to smallest square of control. *p<0.03; **p<0.008. d) Schematic representation of signaling preceding CD69 or Tenascin C expression.

Cell geometry is known to influence the cytoskeletal organization that in turn alters nuclear shape and size [4], [5]. For our experiments, NIH3T3 cells were plated on fibronectin squares of three different surface contact areas- 610, 1450 and 2150 µm^2^. After four hours of incubation, cells were fixed and nucleus stained with Hoechst 33342. Nuclear shape index (NSI) was calculated as a ratio of maximum nuclear area and height of nucleus. This ratio was least for the smallest square and highest for the largest square thus suggesting the impact of cell size and/or shape on nuclear organization (figure 5a, c). Further, we immunolabelled the cells on these different micro-patterns for Tenascin C (TNC), a mechanosensitive protein whose expression can be induced by cell stretching [23]. As expected, the integrated density of TNC staining was highest in the largest pattern and comparable in the intermediate and smaller patterns (figure 5a, c). This suggested to us that gene expression can be modulated by cell geometry and correlates with NSI, as has also been reported previously [8]. Further, to test whether change in nuclear shape alone was sufficient to induce TNC expression, we blocked Erk signaling, since it is known to influence TNC expression [24]. Erk activation was blocked by incubating cells with 25 µM U0126 for 15 minutes, after which cells were plated on micro-patterns in presence of inhibitor. After 4 hours, both control and inhibitor treated samples were stained and imaged (figure 5b). Figure 5c shows the plot for NSI, which is identical to that of control. On the other hand, drug treatment decreased the expression of TNC in all the three sizes of micro-patterns (figure 5b, c). This experiment again highlights that nuclear shape changes alone are insufficient to induce gene expression unless it is coupled with appropriate signaling intermediates.

### Conclusions

In conclusion, our experiments reveal that while nuclear deformation correlates well with gene expression, it alone is insufficient to initiate expression as shown in the schematic in figure 5d. We suggest that nuclear deformation acts in concert with signaling intermediates to the nucleus to alter gene activation. Blocking of signaling molecules such as Erk and NF-κB or depolymerization of microtubules inhibits gene expression, although these molecules do not perturb nuclear elongation. Observations with cytochalasin D treatment lend further support to this point. Cytochalasin D treatment in the M− condition inhibited nuclear elongation and expression of CD69. However, when beads were immobilized in conjunction with cytochalasin D treatment, rescue of nuclear elongation and CD69 expression were observed, suggesting that reduced actin dynamics are also sufficient to initiate signaling. This treatment also showed rescue of Erk phosphorylation, highlighting the significance of nuclear elongation occurring alongside signaling intermediates. Interestingly actomyosin contractility had no effect on nuclear elongation although it did alter the fraction of CD69 positive cells. This is in concert with a recent report that shows that actin polymerization but not myosin IIA was required for assembly of signaling molecules into microclusters and release of Ca^2+^ ions from endoplasmic reticulum stores [25]. Our data also suggests that T-cell activation can be enhanced by immobilization of antigens. This observation is relevant in a physiological scenario where T-cells are mobile but APCs are often immobile. This is also consistent with recent observations of enhanced CD4^+^ T-cell function using antibodies immobilized on substrates with a higher elastic modulus [26].

Our results highlight the fact that nuclear deformation alone cannot bring about changes in transcription programs of cells. The experiments with micro-patterned substrates support the generality of our hypothesis. Inhibition of Erk signaling in NIH3T3 fibroblast cells did not affect NSI in any of the three sizes of fibronectin squares although it did down-regulate TNC expression. Hence, changes in nuclear shape are alone insufficient to initiate gene expression perhaps in various systems. In physiology, cells are constantly subjected to various stresses (for example shear and traction forces) and as a result nuclear deformation is common. Although a number of recent reports suggest coupling between nuclear deformation and gene expression, our results show that deformation without signaling intermediates is insufficient to induce early gene expression. Alterations in nuclear shape may affect the import of regulatory proteins or the spatial positioning of genes allowing them to come in close proximity or go further away. However, it is unclear if nuclear deformation and the extent of deformation is a necessary requirement for gene expression in all multicellular systems.

## Supporting Information

Figure S1 a) Representative image of T-cell stained for pSrc 30 mins post-activation. b) Force calibration curve to calculate the magnitude of force applied on the paramagnetic beads for immobilization. c, d) Fraction of cells that stain positive for CD69 (c) or CD25 (d) in M− and M+ conditions at various time points post-activation.
(PDF)

Figure S2 a) Graph showing the Mean Fluorescence Intensity (MFI) from flow cytometry analysis for the expression of CD69 and CD25 at different time points for M− and M+ conditions (values normalized to M−, mean±s.d. plotted). b) Plot showing the fraction of cells positive for CD69 and CD25 at 3 hr and 6 hr time points, after being exposed to plain beads (M− and M+ conditions) or antigen coated beads (M+ condition). Values plotted are fold change with respect to antigen-coated beads in M−, (mean±s.d.). c) Plot showing the fold change in Thy1.2 positive cells at 3 hr and 6 hr post activation with antigen coated beads in M+ condition. Data was normalized to M− condition (n = 3, mean±s.d.). d) Graph showing percentage of T-cells in contact with antigen-coated beads after 30 minutes of incubation in M− and M+ conditions and their corresponding fraction of CD69 expressing cells at 3 hours (N = 3, mean±s.e.m.). e) Plot showing fraction of CD69 positive cells after activation with antigen coated beads at varying exposure time but constant magnitude of force (∼324 pN) applied on beads (N = 3, mean±s.d.). *p<0.05, **p<0.005. Statistical Significance is calculated with respect to M− (0 mins).
(PDF)

Figure S3 Representative field views from experiments showing the different nuclear morphology seen in control and drug treated cells. Merge of DIC and Hoechst (DNA) image is shown. Blue- DNA; black- antigen-coated beads. Scale bar 5 µm.
(PDF)

Figure S4 a) Graph showing percentage of cells expressing CD69 in control or in presence of transcription inhibitor DRB or translation inhibitor cycloheximide (CHX) in M− and M+ conditions, 3 hour post activation (n = 3, mean±s.d.). **p<0.005. Statistical significance is calculated with respect to the control (M− or M+). b) Graph showing the Mean Flourescence Intensity (MFI) for CD69 expression in absence (D-) or presence (D+) of DRB and with (M+) or without bead (M−) immobilization after 3 hour incubation with antigen coated beads (n = 3, mean±s.d.). c) Quantitative graph showing the levels of CD69 mRNA at the indicated time points in naïve and activated T-cells. Values were normalized to 18S RNA (n = 4, mean±s.d.). *p<0.05, **p<0.005. Statistical Significance is calculated with respect to the M− at the same time point.
(PDF)

Movie S1 Time-lapse imaging of H2B EGFP labeled T-cell activation by surrogate antigen-coated beads in M− condition. Images were recorded at 5 seconds per frame.
(AVI)

Movie S2 Time-lapse imaging of H2B EGFP labeled T-cell activation by surrogate antigen-coated beads in M+ condition. Images were recorded at 5 seconds per frame.
(AVI)
