# Supplementary figures and images for: Role of Actin Dependent Nuclear Deformation in Regulating Early Gene Expression
Source: PLoS One. 2012 Dec 28;7(12):e53031. doi: 10.1371/journal.pone.0053031 (PMC3532443; doi:10.1371/journal.pone.0053031)

a)

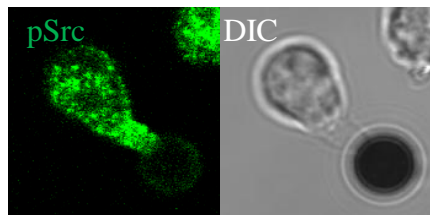

b)

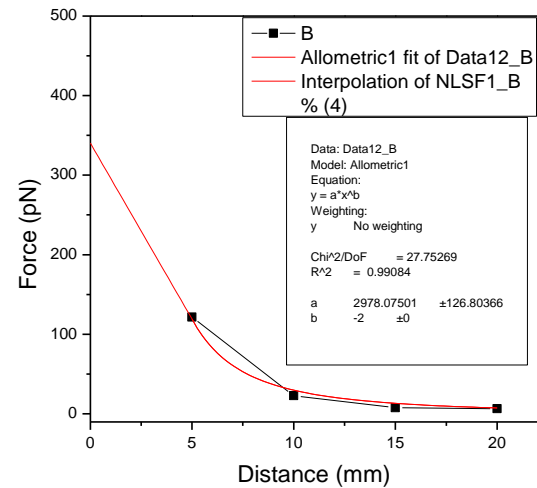

c)

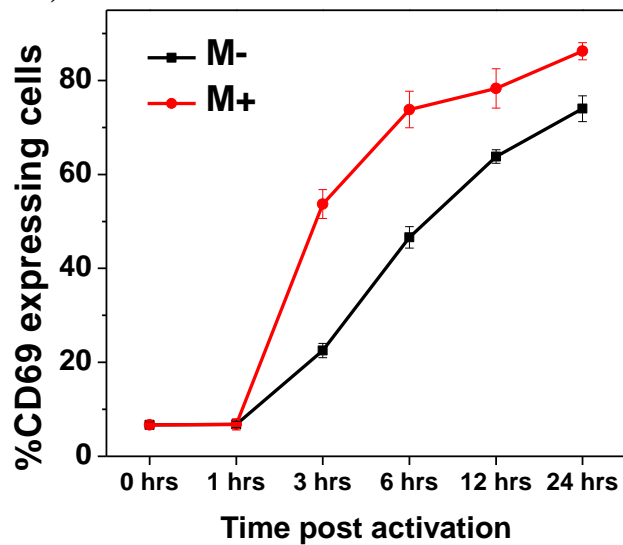

d)

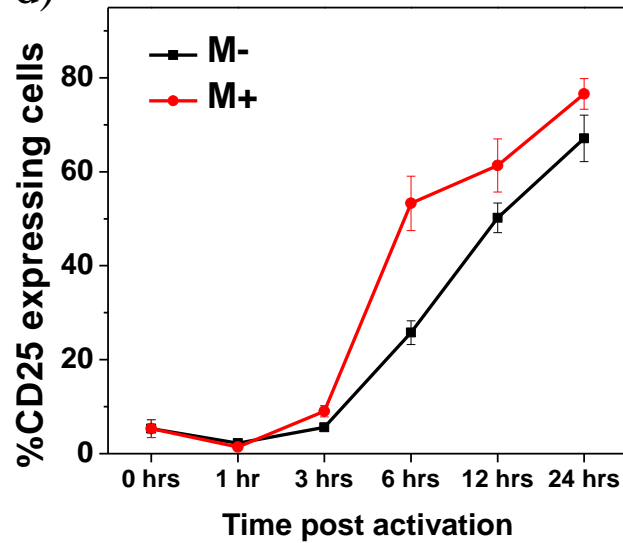

Supplement: Figure S1 — a) Representative image of T-cell stained for pSrc 30 mins post-activation. b) Force calibration curve to calculate the magnitude of force applied on the paramagnetic beads for immobilization. c, d) Fraction of cells that stain positive for CD69 (c) or CD25 (d) in M− and M+ conditions at various time points post-activation. (PDF) [file pone.0053031.s001.pdf]

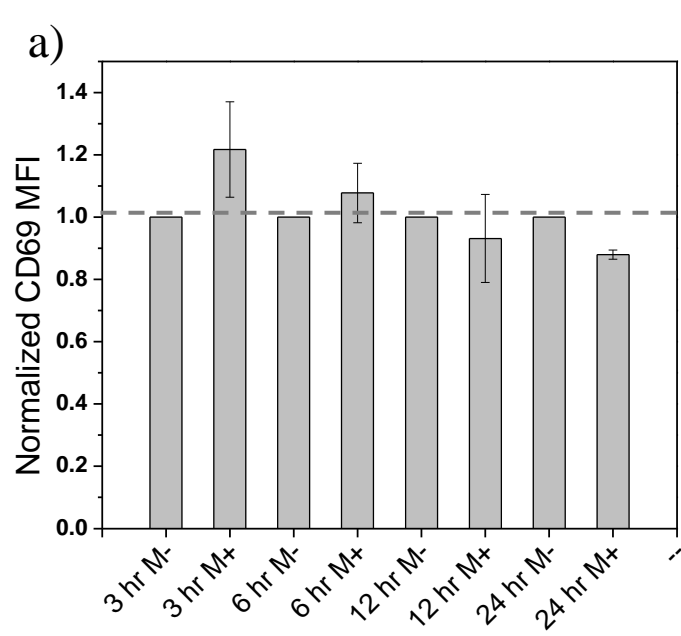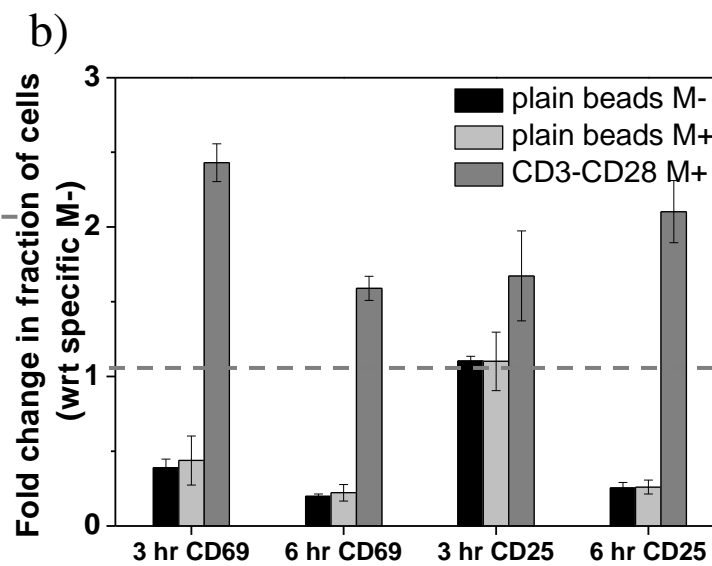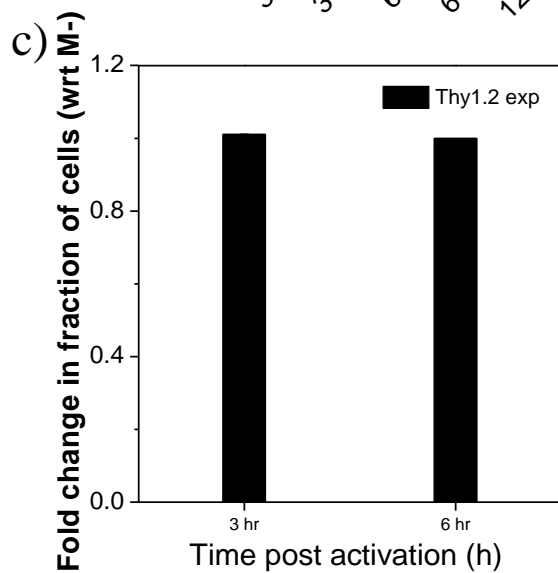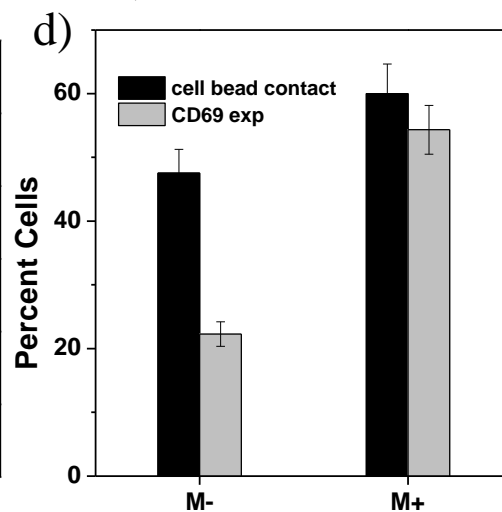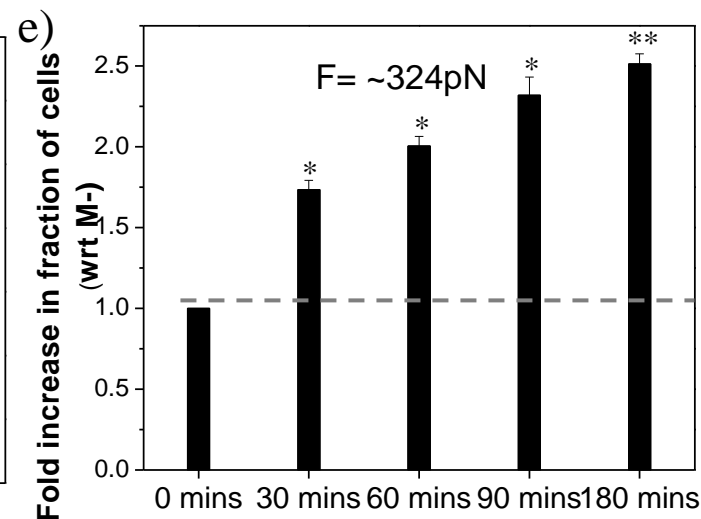

Supplement: Figure S2 — a) Graph showing the Mean Fluorescence Intensity (MFI) from flow cytometry analysis for the expression of CD69 and CD25 at different time points for M− and M+ conditions (values normalized to M−, mean±s.d. plotted). b) Plot showing the fraction of cells positive for CD69 and CD25 at 3 hr and 6 hr time points, after being exposed to plain beads (M− and M+ conditions) or antigen coated beads (M+ condition). Values plotted are fold change with respect to antigen-coated beads in M−, (mean±s.d.). c) Plot showing the fold change in Thy1.2 positive cells at 3 hr and 6 hr post activation with antigen coated beads in M+ condition. Data was normalized to M− condition (n = 3, mean±s.d.). d) Graph showing percentage of T-cells in contact with antigen-coated beads after 30 minutes of incubation in M− and M+ conditions and their corresponding fraction of CD69 expressing cells at 3 hours (N = 3, mean±s.e.m.). e) Plot showing fraction of CD69 positive cells after activation with antigen coated beads at varying exposure time but constant magnitude of force (∼324 pN) applied on beads (N = 3, mean±s.d.). *p<0.05, **p<0.005. Statistical Significance is calculated with respect to M− (0 mins). (PDF) [file pone.0053031.s002.pdf]

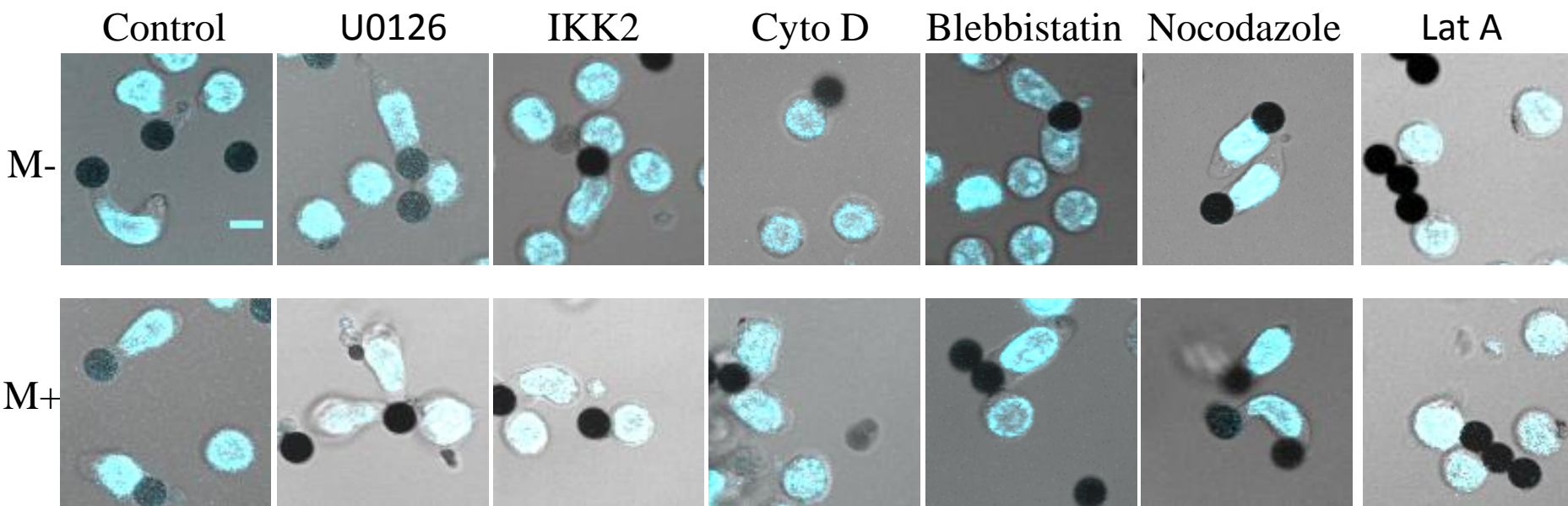

Supplement: Figure S3 — Representative field views from experiments showing the different nuclear morphology seen in control and drug treated cells. Merge of DIC and Hoechst (DNA) image is shown. Blue- DNA; black- antigen-coated beads. Scale bar 5 µm. (PDF) [file pone.0053031.s003.pdf]

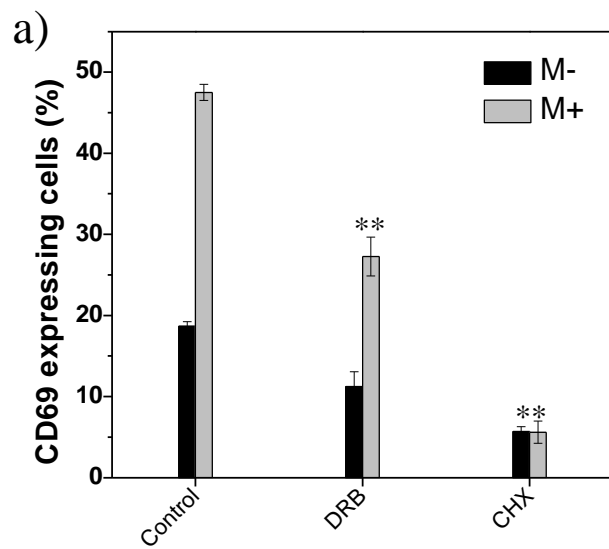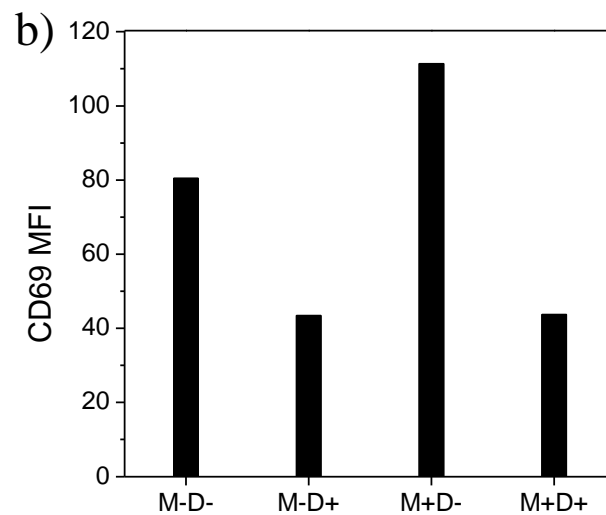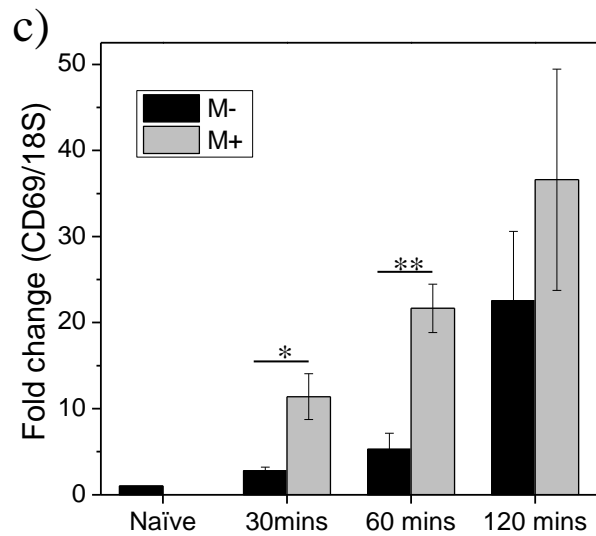

Supplement: Figure S4 — a) Graph showing percentage of cells expressing CD69 in control or in presence of transcription inhibitor DRB or translation inhibitor cycloheximide (CHX) in M− and M+ conditions, 3 hour post activation (n = 3, mean±s.d.). **p<0.005. Statistical significance is calculated with respect to the control (M− or M+). b) Graph showing the Mean Flourescence Intensity (MFI) for CD69 expression in absence (D-) or presence (D+) of DRB and with (M+) or without bead (M−) immobilization after 3 hour incubation with antigen coated beads (n = 3, mean±s.d.). c) Quantitative graph showing the levels of CD69 mRNA at the indicated time points in naïve and activated T-cells. Values were normalized to 18S RNA (n = 4, mean±s.d.). *p<0.05, **p<0.005. Statistical Significance is calculated with respect to the M− at the same time point. (PDF) [file pone.0053031.s004.pdf]
